# Supplementary material for: Inhibitory Effects of Thermolysis Transformation Products of Rotenone on Nitric Oxide Production
Source: Int J Mol Sci. 2023 Mar 23;24(7):6095. doi: 10.3390/ijms24076095 (PMC10093917; doi:10.3390/ijms24076095)
Supplement: Supplementary file 1 [file ijms-24-06095-s001.zip › ijms-2293711-supplementary.pdf]

Supplementary Material

Inhibitory Effects of Thermolysis Transformation Products of Rotenone on Nitric Oxide Production

Gyeong Han Jeong [1,2,†], Hanui Lee [1,2,†], Seung Sik Lee [1,3], Byung Yeoup Chung [1], Hyoung-Woo Bai [1,2,3,*] and Tae Hoon Kim [4,*]

[1] Research division for Biotechnology, Advanced Radiation Technology Institute (ARTI), Korea Atomic Energy Research Institute (KAERI), Jeongeup 56212, Republic of Korea
[2] Center for companion animal new drug development , Korea Institute of Toxicology (KIT), Jeongeup 56212, Republic of Korea
[3] Radiation Biotechnology and Applied Radioisotope Science, University of Science and Technology (UST), Daejeon 34113, Republic of Korea
[4] Department of Food Science and Biotechnology, Daegu University, Gyeongsan 38453, Republic of Korea
* Correspondence: hbai@kaeri.re.kr (H.W.B.); skyey7@daege.ac.kr (T.H.K.)
† These authors contributed equally to this work

Contents

Figure S1. Isolation procedure of thermolysis products of rotenone.

Figure S2. HPLC chromatograms of thermolysis roteonone and the isolated compounds **2**–**6**.

Figure S3. ^{1}H NMR spectrum of compound **2** in acetone-d_6 + D_2O.

Figure S4. ^{13}C NMR spectrum of compound **2** in acetone-d_6 + D_2O.

Figure S5. ^{1}H NMR spectrum of compound **3** in acetone-d_6 + D_2O.

Figure S6. ^{13}C NMR spectrum of compound **3** in acetone-d_6 + D_2O.

Figure S7. ^{1}H NMR spectrum of compound **4** in acetone-d_6 + D_2O.

Figure S8. ^{13}C NMR spectrum of compound **4** in acetone-d_6 + D_2O.

Figure S9. ^{1}H NMR spectrum of compound **5** in acetone-d_6 + D_2O.

Figure S10. ^{13}C NMR spectrum of compound **5** in acetone-d_6 + D_2O.

Figure S11. ^{1}H NMR spectrum of compound **6** in acetone-d_6 + D_2O.

Figure S12. ^{13}C NMR spectrum of compound **6** in acetone-d_6 + D_2O.

Figure S13. Chemical structures of rotenone (**1**) and the degraded products **2**–**6**.

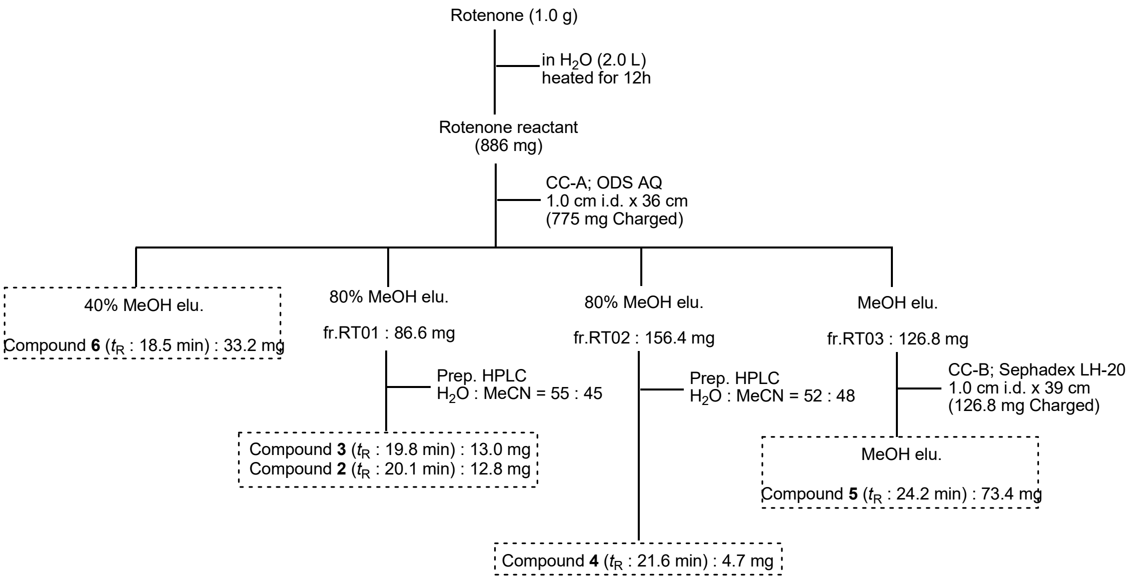

Figure S1. Isolation procedure of thermolysis products of rotenone.

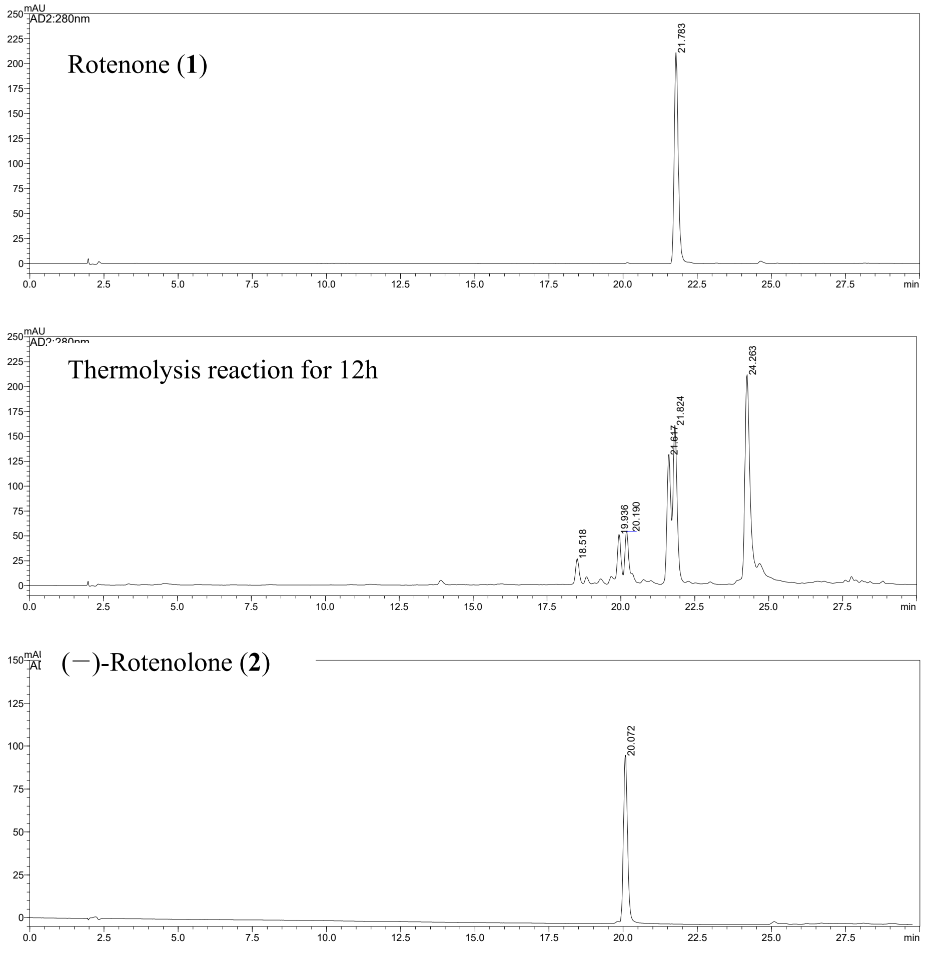

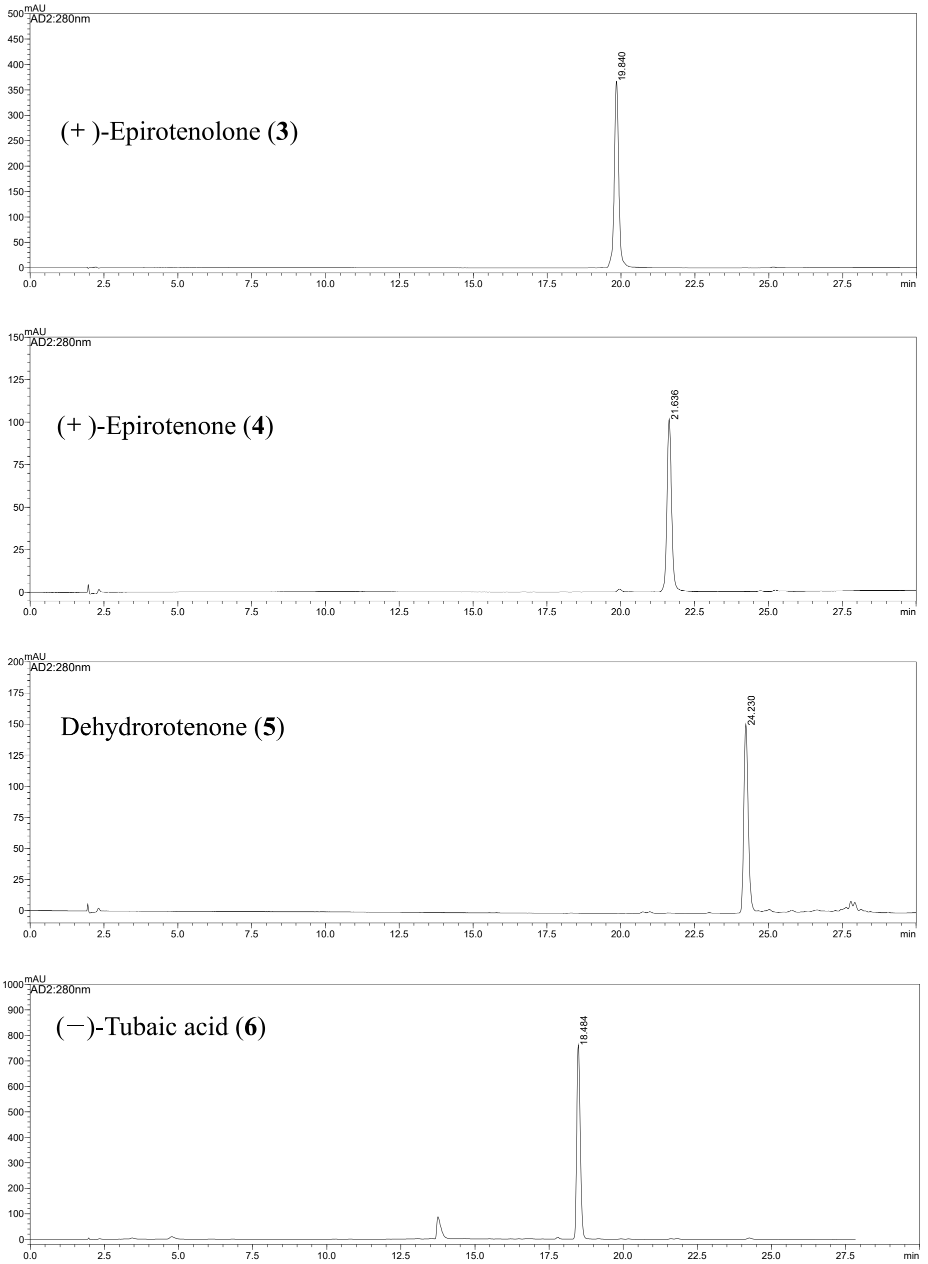

Figure S2. HPLC chromatograms of thermolysis rotenone and the isolated compounds **2**–**6**.

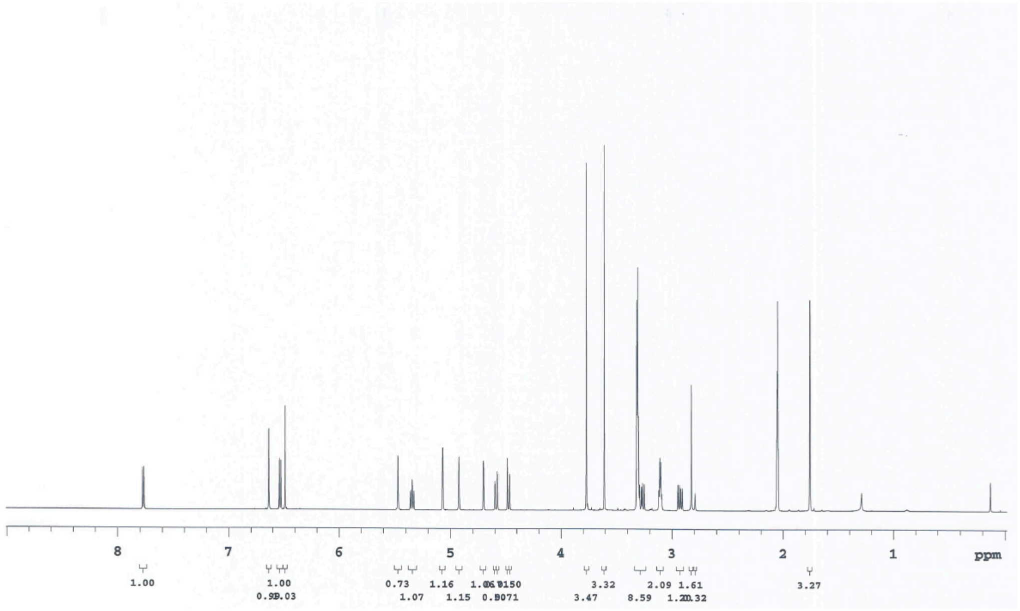

Figure S3. ^{1}H NMR spectrum of compound **2** in acetone-d_6 + D_2O.

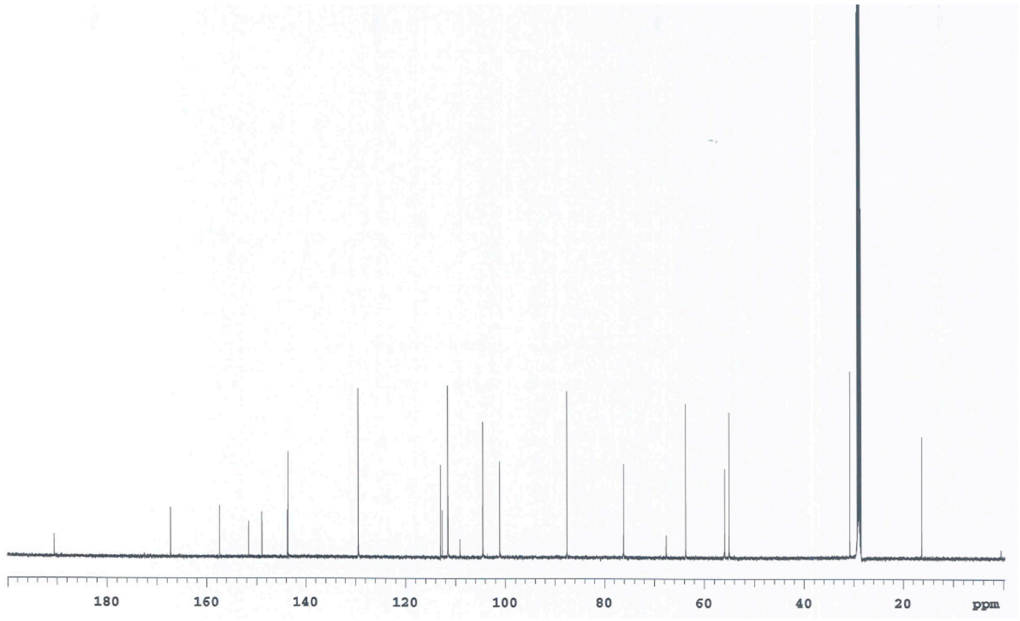

Figure S4. ^{13}C NMR spectrum of compound **2** in acetone-d_6 + D_2O.

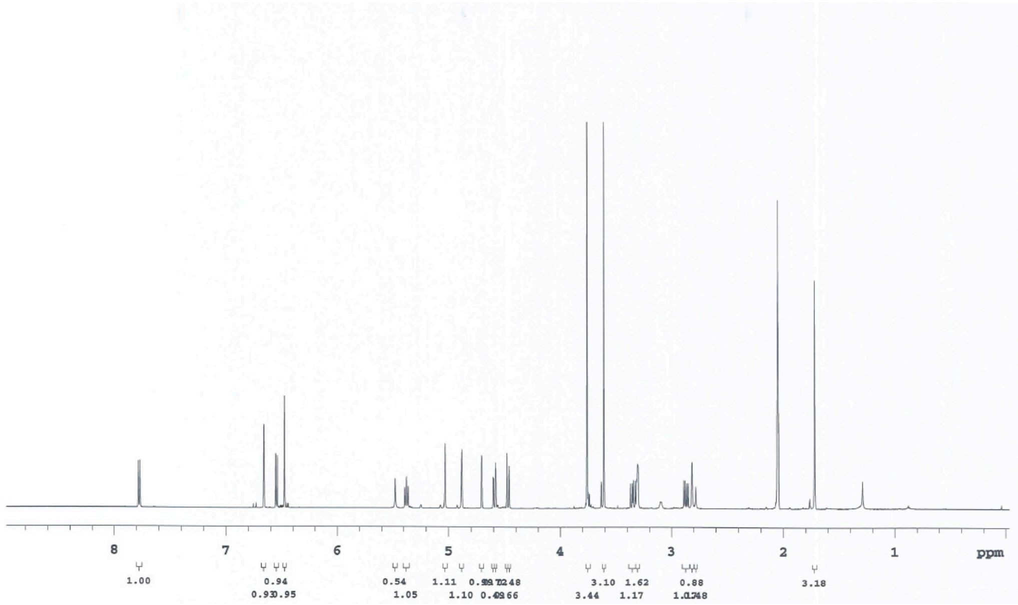

Figure S5. ^{1}H NMR spectrum of compound **3** in acetone-d_6 + D_2O.

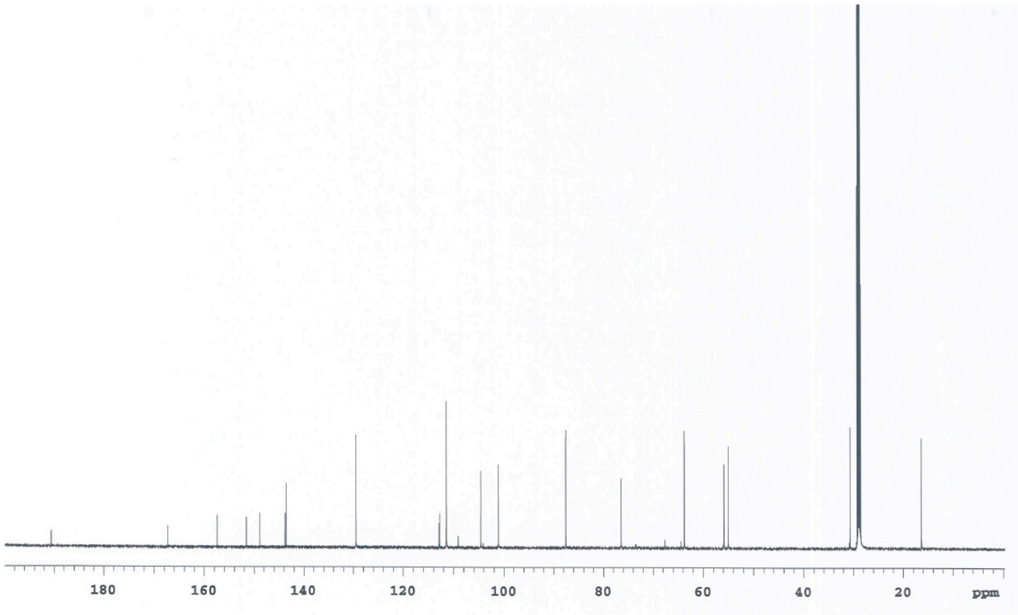

Figure S6. ^{13}C NMR spectrum of compound **3** in acetone-d_6 + D_2O.

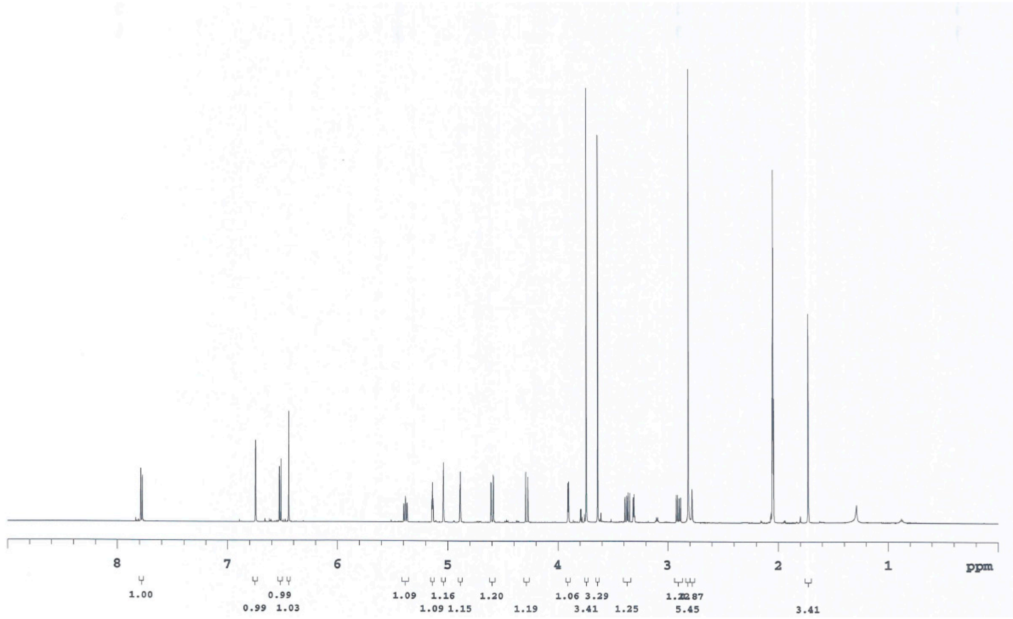

Figure S7. ^{1}H NMR spectrum of compound **4** in acetone-d_6 + D_2O.

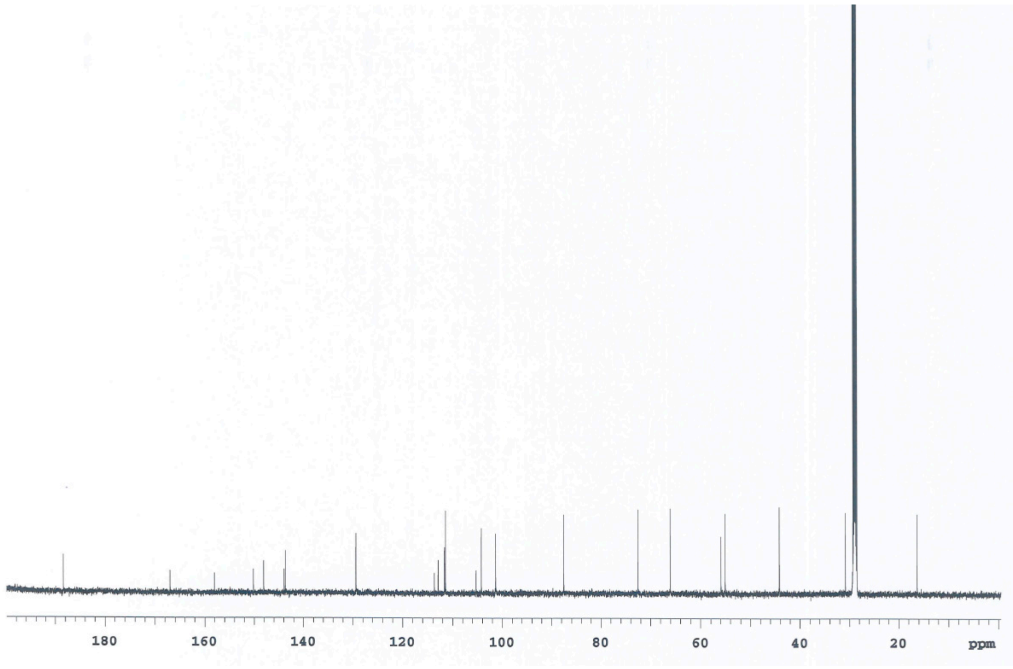

Figure S8. ^{13}C NMR spectrum of compound **4** in acetone-d_6 + D_2O.

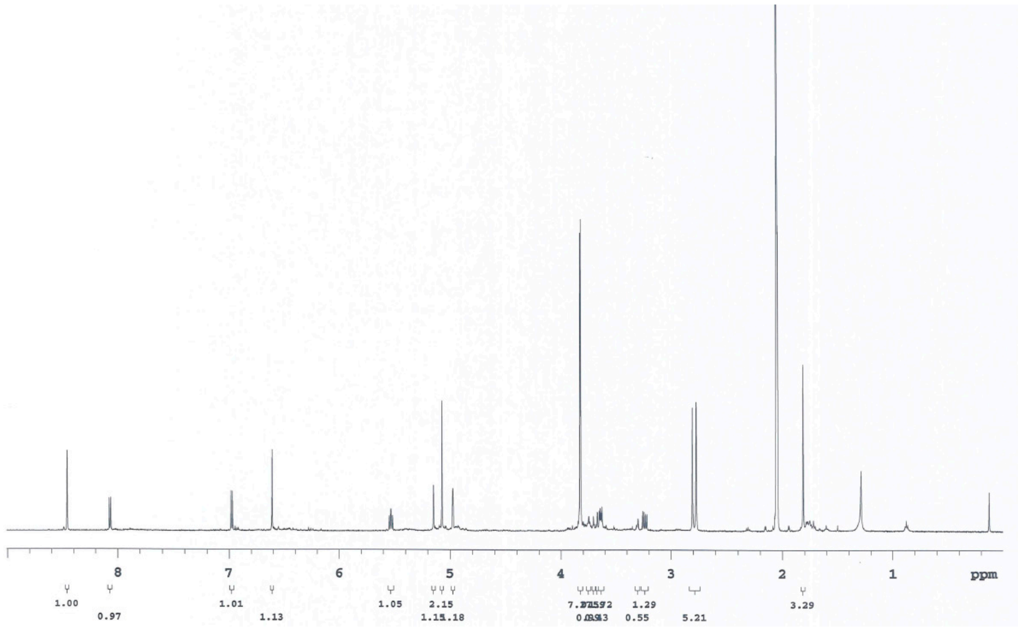

Figure S9. ^{1}H NMR spectrum of compound **5** in acetone-d_6 + D_2O.

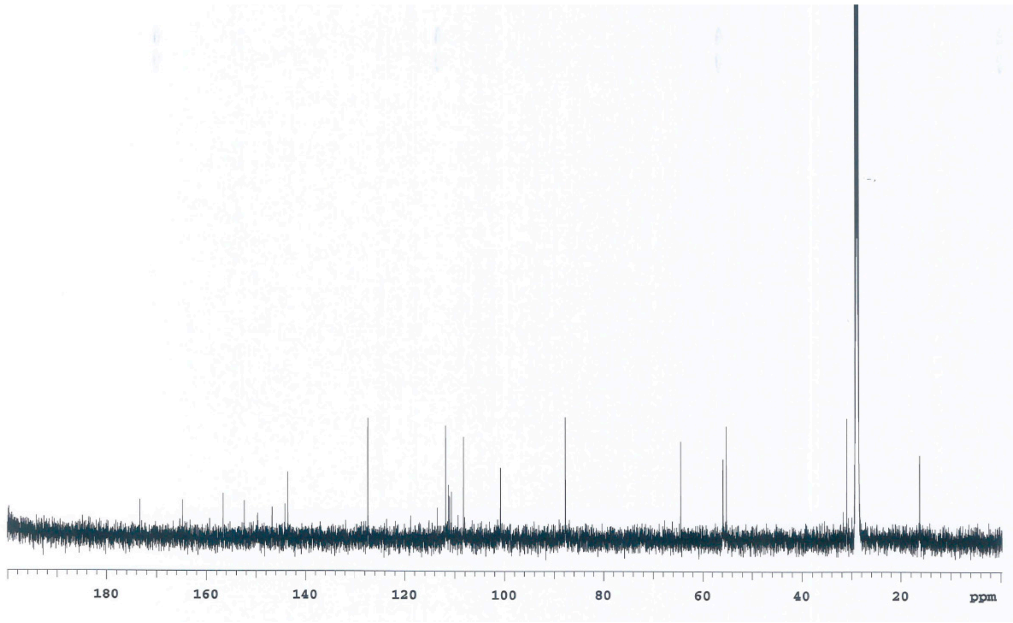

Figure S10. ^{13}C NMR spectrum of compound **5** in acetone-d_6 + D_2O.

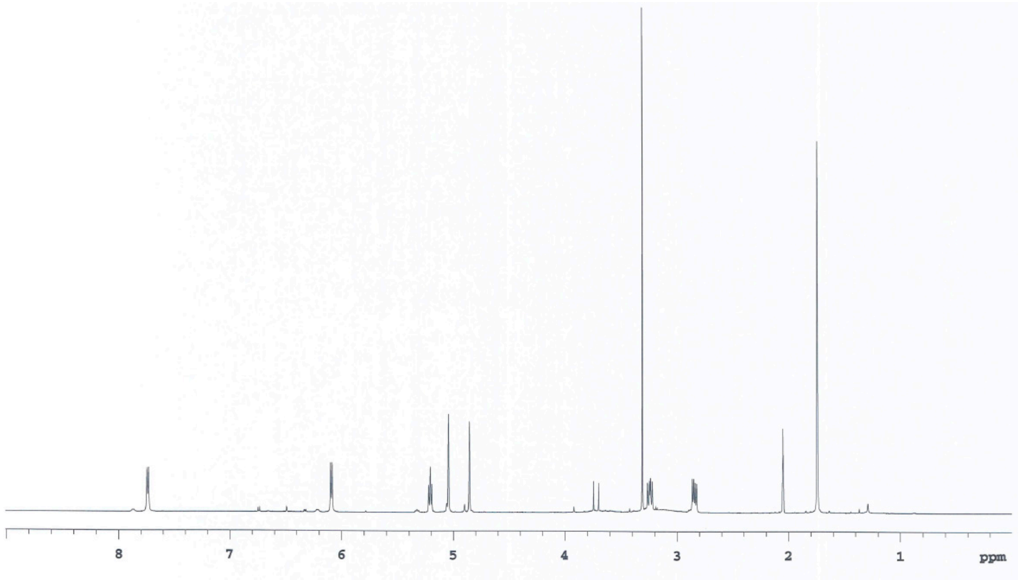

Figure S11. ^{1}H NMR spectrum of compound **6** in acetone-d_6 + D_2O.

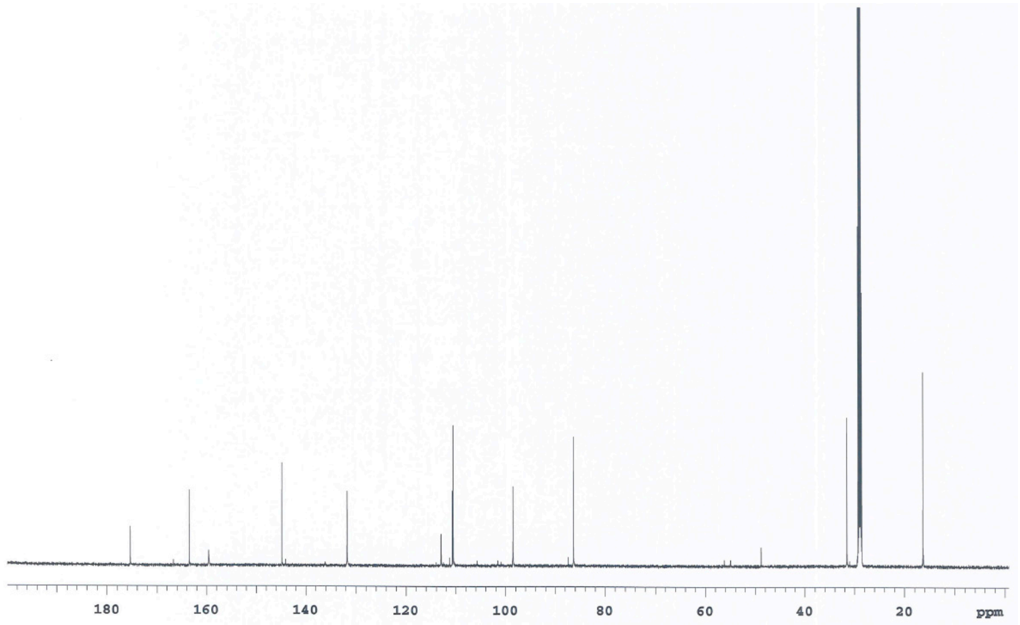

Figure S12. ^{13}C NMR spectrum of compound **6** in acetone-d_6 + D_2O.

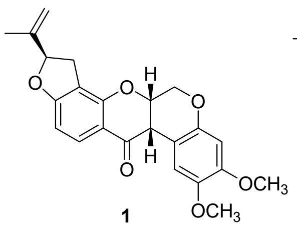

1

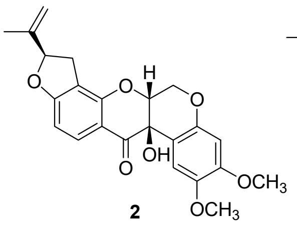

2

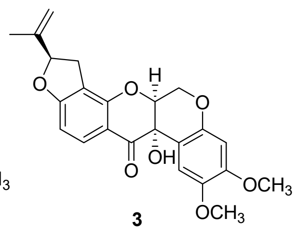

3

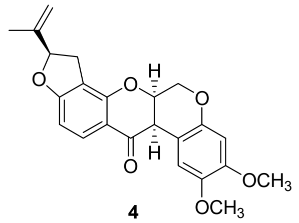

4

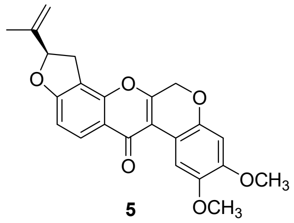

5

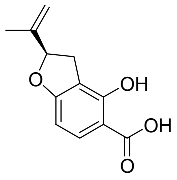

6

Figure S13. Chemical structures of rotenone (**1**) and the degraded products **2**–**6**.
